# The impact of simvastatin intervention on the healing of bone, soft tissue, and TMJ cartilage in dentistry: a systematic review and meta-analysis

**DOI:** 10.1186/s40729-019-0168-4

**Published:** 2019-04-09

**Authors:** Swati Gupta, Massimo Del Fabbro, Jia Chang

**Affiliations:** 1 Department of Periodontology, University of Florida College of Dentistry, Gainesville, FL USA; 2 Department of Biomedical, Surgical and Dental Sciences, Università degli Studi di Milano, Milan, Italy; 3 IRCCS Orthopedic Institute Galeazzi, Milan, Italy

**Keywords:** Statins, Simvastatin, Regeneration, Soft tissue healing, Implants, Periodontal tissues

## Abstract

The review aimed at assessing the osteopromotive potential as well as soft tissue and temporomandibular joint (TMJ) cartilage healing properties of simvastatin by summarizing its efficacy on the current dental treatment of periodontal bone and soft tissue defects, and temporomandibular joint (TMJ) arthritis from the available animals and human studies. An electronic search was performed on MEDLINE, Scopus, and Cochrane Central Register of Controlled Trials (CENTRAL) using a combination of keywords. A hand search was undertaken on seven oral surgery journals. No limitation of publication year in the English language was placed. Controlled randomized animal and human clinical trials, as well as prospective comparative studies, were included. Data on the comparison of topical/systemic simvastatin on bone healing in intrabony and furcation defects, extraction sockets, distraction osteogenesis, as well as soft tissue healing in mucogingival grafting procedures and cartilage protection in TMJ arthritis were extracted from all the eligible studies. Studies with a minimum of ten participants and follow up at least 6 months were included. Ten animal studies and six clinical studies were included in this study. All the animal studies included a minimum of eight sites per group assessed clinically, histologically, and radiographically. All human studies included clinical and radiological evaluation. The results of the review show that simvastatin administration displays positive treatment outcomes in the full range of therapies investigated in the oral regions such as periodontal infection control, periodontal and alveolar bone regeneration, soft tissue grafting, TMJ inflammation reduction, and cartilage repair. Its mechanism includes stimulating bone formation, promoting soft tissue healing, increasing articular and condylar cartilage thickness, as well as reducing inflammation at surgical sites in TMJ disorders. Simvastatin administration is beneficial to the healing of oral bone and cartilage. More studies are desired to determine its potential in soft tissue healing.

## Background

Unlike any other type of tissue, bone and cartilage have a complex morphology and exhibit very limited inherent self-repair potentials. Many studies have suggested that a non-union bone gap greater than 25 nm or even only 500 μm always remains after the primary, non-interventional healing depending on the location, vascularization, and the mechanics [1]. Following tooth loss or extraction, without the mechanical stimulus from teeth, alveolar bone naturally degrades to a significant extent thus complicating prosthetic rehabilitation [2, 3]. When sufficient quantity and quality of the alveolar bone is absent, the interventions of dental implants could be complicated at the site for placement of implants because of impaired osseointegration within atrophic alveolar bone ridge.

Approaches to regenerate bone include specific grafting surgical techniques with autogenous bone, substitutes, barrier membranes, growth factors, stem cell therapy, and lately the osteopromotive pharmacological compounds. The pharmacological approaches have gained popularity owing to their convenience and the advantageous cost-effectiveness when applied with other grafting techniques. Among all the pharmacological compounds, simvastatin has been well investigated since the 90s for its osteopromotive properties. Simvastatin belongs to the family of statins which are structural analogs of HMG-CoA (3-hydroxy-3-methylglutaryl-coenzyme A). Stains could reversibly inhibit HMG-CoA reductase through side chains that bind to the enzyme’s active site and block the substrate–product transition state of the catalyst. They are originally developed to treat hypercholesterolemia. Commercially available -statin medicines include pravastatin, simvastatin, fluvastatin, atorvastatin, cerivastatin, pitavastatin, and rosuvastatin. Of all the statins, simvastatin is one of the most commonly prescribed drugs [4]. The osteoblast-stimulating effects of simvastatin were highlighted by the breakthrough work of Mundy et al. in 1999. They reported that simvastatin could stimulate bone regeneration and promote bone formation in the mouse calvaria defect model [5]. The mechanism simvastatin-mediated bone regeneration could be attributed to its osteoblast promoting, anti-inflammatory, osteoclast inhibiting, and neovascularization properties [6, 7]. The pleiotropic effects of simvastatin in bone metabolism are associated with its induction of BMP-2 and VEGF gene expression to stimulate the differentiation of osteoblastic cells [8]. Meanwhile, simvastatin was found to inhibit bone resorption by reducing expression of TRAP and cathepsin K, preventing the fusion of osteoclast precursors, and decreasing the number of active osteoclasts [9]. Due to those new findings, the dental clinicians investigate how to reposition this drug to treat alveolar bone, oral soft tissue, and TMJ cartilage defects. We summarized the approaches and findings of these works in this paper and found that simvastatin administration displays positive treatment outcomes in the full range of therapies investigated in the oral regions such as periodontal infection control, periodontal and alveolar bone regeneration, soft tissue grafting, TMJ inflammation reduction, and cartilage repair.

A vast literature composed of in vitro experiments, in vivo animal studies, and clinical studies investigated the potential of simvastatin in bone, soft tissue, and cartilage regeneration or protection in the scope of dentistry, with varied results. The purpose of this systematic review was to focus on the effects of simvastatin on healing enhancement in the oral cavity. We aimed to address the following research questions:

1. Does the result vary with the dosage and what is the ideal dose to be used clinically without adverse effects?
2. Is there any difference in outcome with the route of administration—local/ systemic?
3. What is the role of simvastatin in soft tissue healing?
4. What is the reliability of simvastatin in promoting bone formation with or without the adjunct of bone grafts?

## Materials and methods

### Search strategy

This systematic review was reported following the Preferred Reporting Items for Systematic Review and Meta-Analyses statement (PRISMA) [10]. An electronic search was performed on the following databases: MEDLINE, Scopus, and Cochrane Central Register of Controlled Trials (CENTRAL). The last search was performed on December 31, 2017. The search terms used were “simvastatin,” “statin,” “bone healing,” “bone density,” “osseointegration,” “dental implants,” “bone graft,” “grafting,” “periodontal surgery,” “oral surgery,” “extraction socket,” “tooth extraction.” These terms were combined using Boolean operators OR and AND. Furthermore, a hand search of issues from 2000 up to the last issue available on December 15, 2017, including the “Early view” (or equivalent) section was undertaken in the following journals: British Journal of Oral and Maxillofacial Surgery, International Journal of Oral and Maxillofacial Surgery, Journal of Oral and Maxillofacial Surgery, Journal of Periodontology, Journal of Clinical Periodontology, Journal of Periodontal Research, Oral Surgery, Oral Medicine, Oral Pathology, Oral Radiology and Endodontology. The reference list of the retrieved reviews and the included studies was also searched for possible additional eligible studies not identified by the electronic search.

### Inclusion criteria

1. The studies report the results of dental procedures (such as oral surgery, tooth extractions, periodontal treatment, orthodontic treatment, TMJ arthritis therapy, etc.) performed in human and animals, in which simvastatin was topically or systemically used as an adjunct to the standard surgical procedure.

2. The studies provide details on the method of randomization, type and dosage of simvastatin used, the duration of observation, and the report of any adverse effects.

3. The studies compared a test group in which statins were used, versus a control group without using the statins. The use of statins had to be the only difference between the test and control group.

The search was limited to oral surgical procedures in animal and human studies published in the English language only. Restrictions were not placed regarding the publication year. Only prospective studies were included. No limitation on sample size was placed.

### Exclusion criteria

Publications that did not meet the above inclusion criteria and those that were not dealing with original clinical cases (e.g., reviews, technical reports) were excluded. In case of multiple publications relative to subsequent phases of the same study or to enlargements of the original sample size, only the most recent data (those with the longer follow-up and the larger sample size) were considered.

### Selection of the studies

Two reviewers (JC and SG) independently screened the titles and the abstracts of the articles initially retrieved through the electronic search. The concordance between reviewers was assessed using Cohen’s Kappa coefficient. In the case of disagreement, a third reviewer (MDF) was consulted. The full texts of all studies of possible relevance were independently assessed by the same two reviewers to check if they met all inclusion criteria. For articles excluded at this stage, the reason for exclusion was recorded.

### Data extraction

Data were extracted by two reviewers independently (MDF and SG). Cases of disagreement were subject to joint evaluation until an agreement was reached.

The studies were initially categorized on the type of animals used for the research as well as in the kind of procedure performed. The primary variables extracted further from each included study include study design, sample size, type, dosage and administration route of simvastatin, type of oral procedure, associated use of grafting (yes/no), type of graft material, control treatment, jaw (maxilla or mandible), follow-up duration, any outcome variable used to evaluate treatment success, outcomes used to assess radiographic, histological, and histomorphometric bone healing.

The following methodological parameters were also recorded such as the randomization method in randomized studies, the precise definition of outcomes assessment, and the length of the follow-up period for all studies.

### Assessment of the quality of the trial

The methodological quality of the selected studies was evaluated independently and in duplicate by two reviewers (MDF and SG) according to the above methodological parameters. In the case of disagreement, a third reviewer (JC) was consulted. The risk of bias is assessed based on criteria such as randomization, allocation concealment, blinding of the examiner, completeness of follow-up, and the similarity of groups at the start of the study using modified Cochrane Collaboration’s tool for assessment of bias. When all criteria were met, and no more than one criterion was judged unclear, the risk of bias was estimated as low; if two or more criteria were judged unclear and other criteria were met, moderate risk of bias was assigned; when one or more criteria were not met, high risk was assigned. Besides, the assessment parameters like completion and duration of the study, dropouts, and statistical analysis methods appropriateness were determined to declare the study as adequate or inadequate. The authors of the included studies were contacted for providing clarifications or missing information as needed.

### Outcome variables

The primary outcome variables included changes in hard tissue parameters, such as bone/alveolar ridge width and height, probing depth (PD), clinical attachment level (CAL), radiographic defect fill, and bone mineral density. The secondary outcome variables included the assessment of levels of soft tissue inflammatory parameters at the surgical sites.

### Statistical analysis

If two or more comparative studies presented results regarding a similar outcome variable, they were aggregated in a meta-analysis. The weighted mean difference between statin-treated and control group was estimated using a random effect model with the software RevMan (Version 5.3, The Nordic Cochrane Centre, The Cochrane Collaboration, Copenhagen, 2014). Forest plots were produced to graphically represent the difference in outcomes of statin-treated groups and placebo groups for all included studies using defect site as the analysis unit.

## Results

**Fig. 1:**
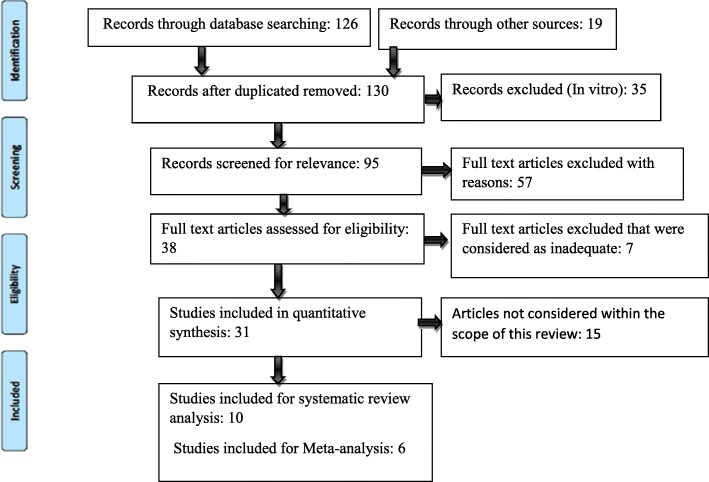
PRISMA flowchart of literature search and screening process

The electronic search yielded 126 articles from the period 2017–2005. Hand searching found an additional 19 articles. After a first screening of the titles and abstracts, 38 articles reporting results of comparative studies that underwent oral surgery procedures in combination with the use of statins were selected. After evaluation of the full-text of these articles, only 16 adequate studies including 10 animal studies and 6 human studies with low risk of bias were considered for review analysis. The meta-analysis was performed with human studies. Animal studies were not considered for quantitative analysis due to the difference in methodologies, protocols, defect types, animal models, and outcome variables. The flowchart summarizing the screening process is presented in Fig. 1.

### Characteristics of the included studies

The characteristics of the included studies are described in Tables 1, 2, and 3. The experimental models used in these studies include rats, rabbits, and beagle dogs. There were a total of ten animal studies. Among them, two studies assessed the effect of systemically administered simvastatin in ovariectomized rats. Vaziri et al. in 2007 determined the most effective dosage of simvastatin (1 × 10^−6^, 1 × 10^−7^, and 3 × 10^−7^ M) for bone formation in ligature-induced periodontitis rat model, whereas Anbinder et al. in 2007 compared the effects of systemic administration of 25 mg/kg of simvastatin with the application of alendronate in both ovariectomized and sham-operated rats for a period of 35 days [11, 12]. Both the studies demonstrated no superior results with simvastatin as compared to controls.

**Table 1:** Summary of the studies on animal models

Author, study type	Procedure and animal type	Group and study type	Key results
Sherif et al. 2016 RCT	20 rats, bilateral extractions	Test group: 2.5% SIM gel Control: no treatment Rats sacrificed at 1st, 2nd, 3rd, and 4th week Buccolingual ridge width measured with bone calipers	Single topical application of 2.5% simvastatin gel improves the quality of the new bone of the healing extraction socket and decreases bone resorption
Wu et al. 2008 RCT	60 rats, extraction	Test group (30): SIM 1 mg/1 ml PLGA scaffold Control: PLGA Rats sacrificed at 1, 2, 4, 8, and 12 weeks. Histology and BMD examination	Higher bone formation rate and quality were found during the extraction socket healing in the experimental group than in the control group at all time points except for 1 week
Vaziri et al. 2007 RCT	49 rats, bilateral ovariectomy	7 groups with ligature placed in all except 1 (sham) Group 1 (*N* = 7), ovariectomy (OVX) plus simvastatin (10–6 M); group 2 (*N* = 7), OVX plus simvastatin (3·10–7 M); group 3 (*N* = 7), OVX plus simvastatin (10–7 M); group 4 (*N* = 7), OVX plus normal saline; group 5 (*N* = 7), OVX group; group 6 (*N* = 7), ligature without OVX; group 7 (*N* = 7), sham surgery without OVX and ligature. Sacrificed after 4 weeks. Radiologic and histologic analysis. Bone loss, attachment loss	Simvastatin inhibits periodontal attachment loss with the least in 10–6 M group. 3·10–7 M had the least effect on the inhibition. Local application of simvastatin shows protective features against the impact of periodontitis on attachment apparatus and alveolar bone
Killeen et al. 2012 RCT Split-mouth study	65 rats, fenestration defects	Test group: 0.5 mg simvastatin in ethanol (SIM-EtOH); 2) 0.5 mg simvastatin in alendronate–cyclodextrin conjugate (SIM-ALN-CD); control group: 3) EtOH alone; 4) ALN-CD alone; or 5) no injections. Sacrificed at 21 days, 48 days. Histometric analysis	Twofold to threefold more new bone width (0.004) was seen in the fenestration defect treatment with the use of systemic ALN after SIM-EtOH injections as compared to local SIM/ALN-CD preparations or short-term SIM-EtOH injections
Kiliç E et al. 2008 RCT	18 rabbits, unilateral distraction osteogenesis	Experimental group I: 2.5 mg/ml of SIM/0.2 g of gelatin sponge applied locally Experimental group II: 10 mg SIM systemically Control: no treatment Sacrificed at 14 days Peripheral quantitative computed tomography, and with histomorphometry	No SSD in the amount of regenerate bone during distraction osteogenesis between the systemic simvastatin group and control group or between the local simvastatin group and control group
Rutledge et al. 2011 Split-mouth study	4 beagle dogs, dehiscence defects bilaterally	Local placement of porous HA-collagen grafts with resorbable membranes with or without 10 mg SIM followed by local injections. Sacrificed after 2 months Histomorphometry	Locally injected SIM can induce modest amounts of new bone formation within the dehiscence defects in closed injection sites over a periosteal surface
Ozec et al. 2007 RCT	23 rats, critical-sized defects in the mandibles	Experimental group: 2.5 mg/Ml SIM mixed with 0.02 g of gelatin sponge. Passive control Active control: gelatin sponge mixed with water Sacrificed at day 14 Radiology and histology assessment	New bone formation and density of new bone in mandibular defects are more significant in the experimental group than control groups
Anbinder et al. 2007 RCT/Split mouth	54 rats, two groups: ovariectomized (OVX) or sham operated	Experimental group: simvastatin (SIN–25 mg/kg), Active control: sodium alendronate (ALN–2 mg/kg) or Passive control: water (control) orally. Sacrificed after 35 days Radiographic bone density measured	No SSD in alveolar bone formation between ALN and SIM group
George MD et al. 2013 RCT	32 rats, randomized 5 groups. TMJ inflammation induced	I: Controls II: ETH III: 0.1 mg SIM, 3) IV: 0.5 mg SIM, V: 0.15 mg TH. Time: 28 days H&E	SIM & TH reduced the TMJ articular layer thickness, 0.5 mg decreased inflammation
Holwegner et al. 2015 RCT	44 mature rats CFA induced inflammation in right TMJ 6 groups	I: CFA + 0.5 SIM II: CFA + EtOH III: CFA + 0.15 TH IV: CFA + SIM + H V: CFA VI: Control (left) Time: 4 weeks CT, BV, BMD	CFA combination groups: TMJ ramus height > than CFA alone BV:CFA + 0.5 SIM > CFA + SIM + TH Condylar width, bone density: least in steroid grp as compared to SIM

*SIM* simvastatin, *HA* hydroxyapatite, *TH* triamcinolone hexacetonide, *CFA* complete Freund’s adjuvant, *EtOH* ethanol

**Table 2:** Summary of human studies

Author	Procedure type	Group and study type	Key results
Gouda et al. 2017 Bilateral RCT	6 patients 8 sinus lifts in sites with < 8 mm available bone. CBCT at 1 week and 9 months to determine the change in bone height and %age of bone loss. Histomorphometry to determine new bone formation rate	Experimental group: 7.21 mg simvastatin/1 g beta-TCP Control: beta-TCP	SSD in new bone formation of maxillary sinus bone grafting. More in the SIM group. No SSD in the amount of bone loss between both groups
Ranjan et al. 2017 RCT	20 patients, 40 bilateral periodontal intrabony defects. Open flap debridement. Clinical and radiographic outcomes	Experimental group: OFD + 1.2 SIM compared to Control: OFD + placebo. GI, PI, PD, CAL at 3, 6, 9 months. Bone fill assessment	SSD decrease in PD. GI and increase in CAL in the experimental group. No SSD in PI. SSD increase in the amount and percentage of bone fill in the experimental group
Kinra et al. 2010 Bilateral RCT	15 patients Bilateral 2-walled or 3-walled periodontal intrabony defects. Regenerative periodontal therapy with bone grafts. Clinical and radiographic outcomes	Experimental group: DFDBA and SIM (10–8 M) Control group: DFDBA PI, PD, intrabony pocket depth at 10, 24 weeks	SSD in an increase in bone fill, CAL gain, reduction in PD in the experimental group
Chauhan et al., 2015 Bilateral RCT	30 patients Bilateral impacted mandibular third molar extraction sockets. Extraction and site preservation. Clinical and radiographic outcomes	Experimental group: Gelfoam with 10 mg simvastatin Control: Gelfoam Pain, swelling, bone density with software at 3 months	No SSD in facial swelling and pain between both groups. SSD increase in bone density (calculated by gray level histogram) in the experimental group
Pradeep et al. 2012 Bilateral RCT	72 patients Mandibular molar buccal Class II furcation defects. Non-surgical periodontal therapy. Radiographic assessment of (PD), (RVAL) vertical and horizontal (RHAL) attachment levels	Group I: SRP plus placebo Group II: SRP plus 1.2-mg SIM Recall at Baseline, 3 and 6 months	SSD in the experimental group < control SSD in PD in the experimental group at all periods. SSD increase in bone fill, RVAL, RHAL in the experimental group
Madi M, Kassem A 2018 RCT	*N* = 40 Free gingival graft procedure. Visual analog scale for pain and discomfort scoring. Wound healing score	4 groups: group I: Simvastatin suspension (S), group II: simvastatin/chitosan gel (SC), group III: chitosan gel (C), group IV: petroleum gel (P). Recall at 1, 3, 5, 7, 14 days	SSD in VAS and wound healing score at 3, 7, 15 days in the group II, simvastatin/chitosan gel (SC) application

*DFDBA* demineralized freeze-dried bone allograft, *TCP* tricalcium phosphate, *OFD* open flap debridement, *SSD* statistical significant difference, *GI* gingival index, *PI* plaque index, *PD* probing depth, *CAL* clinical attachment level, *SRP* scaling and root planning, *SIM* simvastatin, *VAS* visual analog scale

**Table 3:** Characteristics of the included studies

Included studies	Clear inclusion and exclusion criteria	Randomization method	Assessment parameters (two or more)/validated measurements	Duration of study	With/without carrier and route	Risk of bias
Vaziri et al. 2007	✓	No	2	4 weeks	No carrier/injection	Low
Killeen et al. 2012	✓	No	1	48 days	Systemic/local injection	Low
Kilic et al. 2008	✓	No	2	14 days	Systemic/local	Low
Rutledge et al. 2011	✓	No	1	60 days	Local injections	Low
Ozec et al. 2007	✓	No	2	14 days	Systemic	Low
Sherif et al. 2016	✓	No	1	4 weeks	Topical	Low
Wu Z et al. 2008	✓	No	2	12 weeks	Local	Low
Anbinder et al. 2007	✓	Yes	1	35 days	Oral	Low
George MD et al. 2013	✓	No	1	30 days	Injections/carrier	Low
Holwegner et al. 2015	✓	No	2	28 days	Injections/carrier	Low
Gouda et al. 2017	✓	No	2	9 months	Local	Low
Ranjan et al. 2017	✓	No	2	9 months	Local	Low
Kinra et al. 2010	✓	No	2	24 weeks	Local	Low
Chauhan et al. 2015	✓	No	2	3 months	Local	Low
Pradeep et al. 2012	✓	No	2	6 months	Local	Low
Madi and Kassem. 2018	✓	No	2	14 days	Topical	Low

Wu et al. 2008 and Sherif et al. 2016 assessed the extraction and site preservation effect of 1 mg/ml of simvastatin in PLGA gel and 2.5% simvastatin in 1 ml chitosan gel respectively [13, 14]. Both studies concluded that simvastatin is potential to preserve bone height and bone mineral density (BMD). Increase in bone density was reported at the periods between 1 and 4 weeks, while the increase in bone height was only evident at 8 weeks [13]. Killeen et al. in 2012 conducted a study comparing the effects of 0.5 mg of simvastatin with 0.5 mg simvastatin in alendronate-cyclodextrin conjugate on fenestration defects created on molar roots in a rat model [15]. The study highlighted the osteopromotive impact of local simvastatin when used in conjunction with systemically administered alendronate. Rutledge et al. in 2011 compared simvastatin (10 mg) with porous hydroxyapatite collagen sponges applied locally as well as injected in dehiscence defects in dogs. The study demonstrated the ability of simvastatin to induce bone growth at sites of thin bone and edentulous sites [16]. Another study by Ozec et al. in 2007 compared simvastatin (2.5 mg/ml) with gelatin sponges for bone density in critical-sized defects in rats [17]. Healing in closed defects like distraction osteogenesis was determined by Kilic et al. in 2008 in rabbits. The experimental group included simvastatin administered locally in the dose of 2.5 mg/0.2 g of gelatin and 10 mg administered systemically [18].

The human studies included in this analysis showed greater homogeneity in the methodology than the animal studies [19–24]. Therefore, we performed a meta-analysis on these six selected human clinical studies. Of all these six human studies, only one study investigated soft tissue healing potential of simvastatin. All these clinical trials aimed to determine if simvastatin intervention could benefit surgical approaches and improve clinical and radiological parameters. The dosage of simvastatin used in all studies was 1.2 mg except in the study by Gouda et al. in 2017 [23]. In this study, the researchers conducted a split-mouth study comparing 0.1 mg of simvastatin/14 mg of beta-TCP to beta-TCP in maxillary sinus lifts. Although this study included the least number of patients among all the selected studies in this review, it exhibited the strength in extending the follow-up to 9 months after the surgeries.

The parameters of all selected studies included the changes in probing depth and clinical attachment levels. Some studies evaluated bone density (gray levels) and bone fill. One study investigated soft tissue parameters. Among them, Kinra et al. in 2010 compared simvastatin (10^−8^ M/mg of DFDBA) to DFDBA in bilateral two-walled and three-walled defects [19]. Ranjan et al. in 2017 assessed bone formation in intrabony defects by comparing open flap debridement (OFD) and a placebo to OFD with 1.25% simvastatin in the defects [20]. Pradeep et al. in 2012 performed a 6-month prospective study on 72 patients and compared scaling root planning (SRP) to SRP in combination with 1.2 mg simvastatin in class II furcation defects. This study included the highest patient number and the longest follow-up period [21]. Chauhan et al. in 2015 determined the site healing pattern in extraction sockets with 10 mg simvastatin. It also assessed the effects of simvastatin on patients’ comfort, post-op swelling, and pain [22]. Impact of statins on cartilage healing has also been studied in intra-articular-induced arthritis models in rats [37, 38]. The studies suggested that 0.5 mg of simvastatin has the potential to reduce sub-synovial inflammation and inducing new bone formation in steroid-induced bone resorption sites.

### Results of analysis for bone height in animal studies and meta-analysis for CAL in human studies

Two animal studies assessed the effect of simvastatin on the bone height in extraction sockets [11, 13], and one evaluated the changes of bone height in the ligature-induced periodontitis with simvastatin treatment [14]. Even though all these studies suggested that simvastatin may have a beneficial effect, due to consistent differences in the protocols, outcome variables, and evaluation methods among these studies, it was not feasible to estimate an overall effect with meta-analysis.

**Fig. 2:**
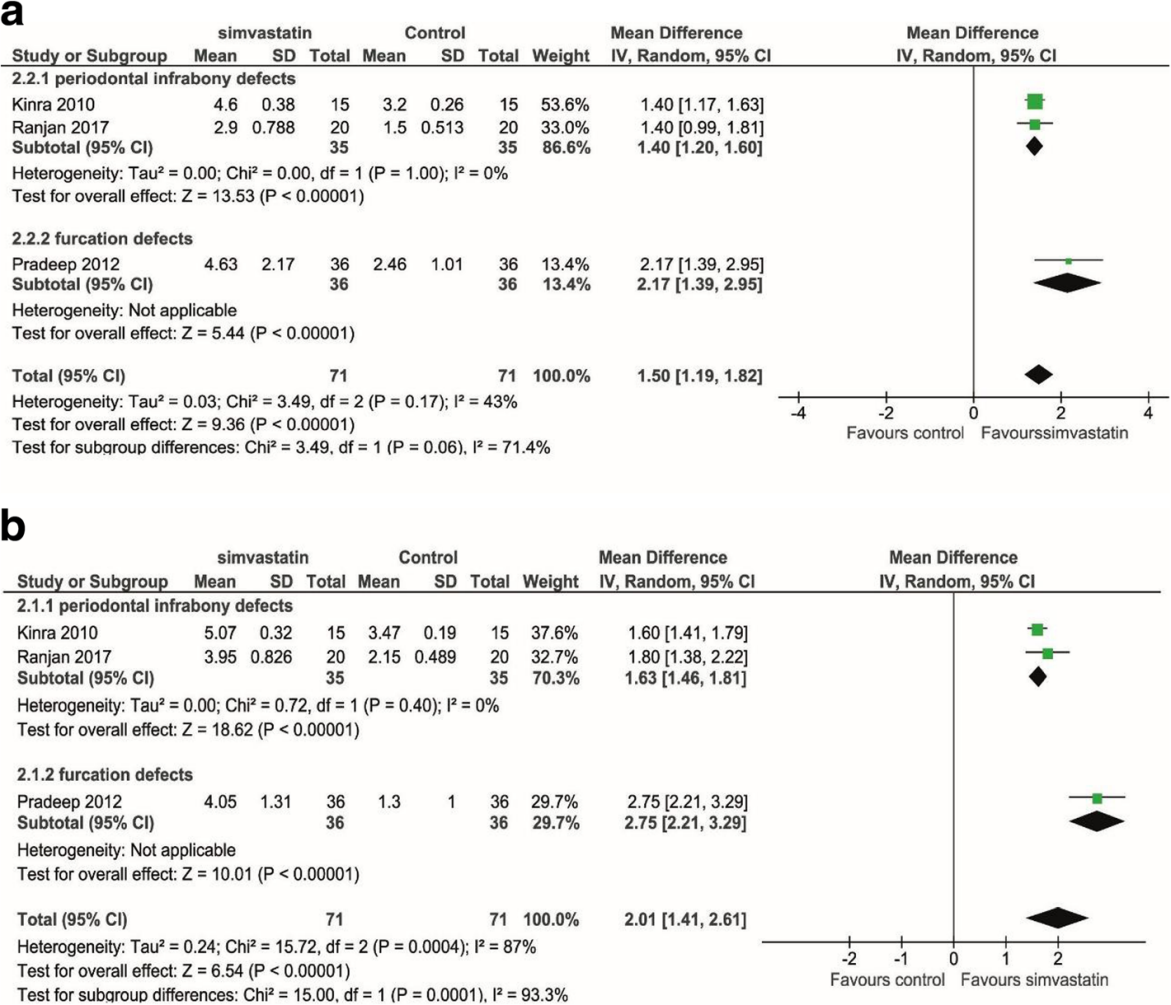
Meta-analysis using a random effect model for assessing bone height changes in various types of defects in human studies. **a** Clinical attachment level (CAL) changes at 6 months. **b** Probing depth (PD) reduction. Significant positive effect of simvastatin was found in both cases. Heterogeneity was found for PD (*I*^2^ = 87%, *p* = 0.0004), but not for CAL (*I*^2^ = 43%, *p* = 0.17). Mean differences and 95% confidence intervals are expressed in mm

Three human studies assessed the changes in CAL (Fig. 2a) and PD (Fig. 2b) in periodontal defects [19–21]. There was evidence of a significant benefit of simvastatin for both CAL gain (mean difference 1.50 mm, *p* < 0.001, 95% CI = 1.19, 1.82 mm) and PD reduction (mean difference 2.01 mm, *p* < 0.001, 95% CI = 1.41, 2.61 mm). All the studies favored the statin-treated group. The study by Gouda et al. [23] also showed a significant positive effect of statin on bone height improvement in the maxillary sinus augmentation. But we did not perform a meta-analysis on these topics because it is the only study on such procedure.

### Results of analysis for bone width in animal studies and meta-analysis for defect bone fill in human studies

Four animal studies investigated the changes in bone fill after dental treatment with the use of simvastatin [14–16, 18]. Among these studies, one study was conducted on dehiscence [16], one on fenestration defects [15], one study was on tooth extraction defects [14], and one on distraction osteogenesis [18]. Except for the study on fenestration, all the other studies showed a positive effect of simvastatin, although no statistical significance was reached in two studies [16, 18]. Due to heterogeneity in the protocols and outcome measures, no meta-analysis could be performed to summarize the results of these studies.

**Fig. 3:**
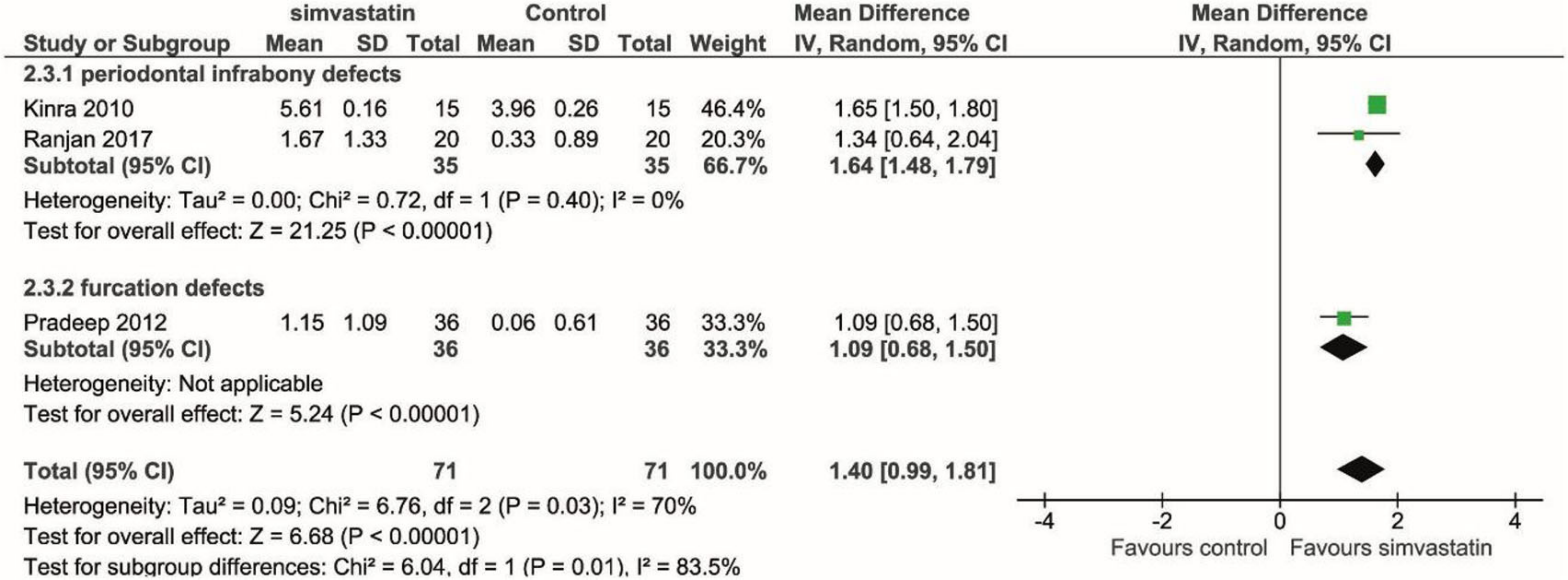
Meta-analysis using a random effect model for assessing bone fill in various types of defects in human studies. Overall analysis showed a significant positive effect of simvastatin in enhancing bone defect fill (*p* < 0.0001). Slight heterogeneity among studies was found (*I*^2^ = 70%, *p* = 0.03). Mean differences and 95% confidence intervals are expressed in mm

Three human studies assessed the changes in the width of alveolar ridge evaluating bone fill radiographically [19–21]; we found the significant improvement of alveolar ridge width in the simvastatin group than its control group (mean difference = 1.40 mm, *p* < 0.001, CI = 0.99, 1.81 mm) (Fig. 3).

### Results for analysis for bone mineral density in animal studies

Five animal studies investigated whether simvastatin application alters bone mineral density (BMD) [11–13, 17, 18]. Vaziri et al. in 2007 measured bone matrix resorption in ovariectomized rats using a dental X-ray unit, and giving densitometric measurement values in mm [11]. Anbinder et al. in 2007 measured BMD in ovariectomized rats with a digital dental X-ray unit, giving optical density values in gray levels [12]. Wu et al. in 2008 measured BMD with dual-energy X-ray absorptiometry (DXA) and provided the results in the unit of mg/cm^2^ [13]. Kilic et al. in 2008 measured BMD changes in rabbits by peripheral quantitative computed tomography (pQCT) and represented the results in a Hounsfield scale [18]. Ozec et al. in 2007 were excluded from the final analysis because this study did not provide sufficient data [17]. These four studies all showed the mild positive effect of simvastatin on BMD, but without the statistical difference. Due to the methodological differences, it was not feasible for us to aggregate the results of BMD changes using a meta-analysis.

## Discussion

Simvastatin was repositioned to anti-inflammatory and osteopromotive purpose recently. Researchers found that simvastatin could accelerate bone regeneration and soft tissue healing by increasing osteoblastic differentiation and stimulating neovascularization via its influence on bone morphogenetic proteins and endothelial growth factor [24, 25]. Statins may also show the anti-inflammatory effect by inhibiting the tissue degrading enzymes like matrix metalloproteinase (MMPs) in rheumatoid arthritis [26]. In the present review, we want to summarize the application of simvastatin in the scope of dental treatments including periodontal regeneration, bone grafting in tooth extraction sockets, distraction osteogenesis, soft tissue healing after mucogingival grafting, and TMJ arthritis therapy. The results of the present systematic review showed that simvastatin has a positive effect on bone regeneration, soft tissues healing, and TMJ articular cartilage healing. Such clinical findings are consistent with the statins’ biological function [27]. For example, the significantly higher alveolar bone level/amount and attachment level in periodontitis patients with simvastatin intervention could be contributed to its synergistic effect of reduced anti-inflammatory response, less soft and hard tissue degradation, and enhanced wound healing potential.

The dosage of simvastatin for dental osteogenic purpose varied from as low as 1.2 mg applied locally to 10 mg/kg/day administered systemically. Interestingly, the anti-inflammatory and osteopromotive properties of simvastatin intervention are related to its dose. Wang et al. reported that 5–10 mg/kg/day applied locally in the fracture region supported bone healing [28]. High dose of simvastatin (20 mg/kg/day) increases bone formation, whereas low dose (1 mg/kg/day) decreases bone formation and induces bone resorption [29]. Two human trials in this systemic study showed 0.1 mg simvastatin/14 mg beta-TCP in tooth extraction sockets, and 10 mg simvastatin/2 ml gel foam applied in maxillary sinus lifts increased bone formation [22, 23]. Gutierrez et al. in 2006 determined that the topical application of statin was 50 times more efficient to promote bone formation than the oral administration [30]. While Kilic et al. in 2008 reported no significant difference between the local and systemic application of simvastatin in enhancing bone density in a model of distraction osteogenesis [18]. Rutledge et al. in 2011 reported a 240% increase in bone density with the local application of simvastatin in mandibular defects [16]. However, Kilic et al. reported the bone surface area was higher in the local application group, although it did not reach statistical difference. Overall, timing, dose, administration manner, and schedule of simvastatin are all important factors to be considered how to perform simvastatin intervention in dentistry. From the researches as mentioned earlier, 0.5–1 mg of simvastatin falls within safe limits for intraoral topical applications. The dose of simvastatin to treat the systemic bone loss such as osteopenia or osteoporosis was examined by several studies. Ho et al. in 2009 showed that simvastatin (20 mg/kg/day) enhances bone formation by increasing osteoblast numbers and osteogenic protein expression in ovariectomized rats [31]. This was proved by the increased bone density, bone height, and better bone defect healing by Vaziri et al., Anbinder et al., and Junqueira et al.’s study [11, 12, 32]. However, Anbinder et al. reported no statistical difference was found when systemically administered 25 mg/kg/day simvastatin with alendronate (2 mg/kg/day) in ovariectomized rats [12]. Therefore, based on all currently available studies, we cannot draw a clear conclusion about the dose and intervention pathways for either local or systemic administration of simvastatin for our dental treatment. More studies are required to optimize its clinical administration.

Simvastatin is a lipophilic drug. Hence, to increase its absorption at the local sites and to delay the release of drug thereby achieving a sustained release, simvastatin is usually mixed with carriers like ethanol, chloroform, and methanol [22], or mixed it with bone graft materials such as DFDBA and beta-TCP [19, 23]. Nyan et al. in 2009 and other research explained the burst release phenomenon of simvastatin from the graft particles on the first day followed by slow release. This is advantageous as the optimal dose of the drug stimulates local cells to express BMP-2 without inducing the inflammatory reaction [33–36]. Another reason to use carriers is for space maintenance, for which thermosensitive materials are usually considered. These materials change their form from liquid to semi-solid, which aids in retaining the drug at a specific site for a longer duration of time. Hence, one of the aims in the present systematic review was directed at determining the outcomes after the administration of simvastatin with and without a carrier. Studies by Sherif et al., Holwegner et al., and George et al. [14, 37, 38] reported no statistically significant differences in their results between the two groups. Rutledge et al. and Ozec et al. [16, 17] have used gel foam and collagen sponges as carriers with simvastatin to determine, if any advantage was obtained, with the use of respective compounds. Rutledge et al. [16] in his study reported that the use of ethanol prevents the backflow of the drug compared to carriers like methylcellulose due to their thermosensitive property. Also, in the same study, they compared the effects of simvastatin on bone formation with or without the adjunctive use of bone grafts. Though they observed a significant difference in the bone formation between the two groups, the results were not statistically significant. Sherif et al. [14] in his paper further commented that there is no definite consensus on the indications for the use of carriers and it is determined by the type of surgical procedure.

In the present review, we also review the study investigating simvastatin intervention on the healing of articular cartilage. Two recent studies determined the outcome of simvastatin injections on experimental temporomandibular arthritis in rats. In one of the studies, a dose of simvastatin compared was 0.1 and 0.5 mg, whereas in the other study, 0.5 mg of simvastatin was compared with the 0.15 mg of triamcinolone hexacetonide (steroids) injections [37, 38]. The period for assessment was 28 days in both. Both the studies concluded by agreeing on the anti-inflammatory property of simvastatin. It was also suggested that simvastatin could preserve normal condylar growth of cartilaginous tissue, if not additive to the present extension. Also, 0.5 mg was found to be better than 0.1 mg in reducing retrodiscal inflammation which collaborates with the previous studies on the comparison of various doses of simvastatin. The study had recorded that 0.1 mg was associated with the least inflammatory reaction. However, there was increased bone formation and anti-inflammatory effect with 0.5 mg simvastatin [33, 39].

The soft tissue healing potential of statins has also been established in a very recent study by Madi and Kassem [40]. The study assessed palatal healing with simvastatin and chitosan combination (10 mg/ml) after free gingival graft procedure. A visual analog scale determined pain discomfort at various time intervals up to 14 days. Pain scores were comparatively reduced with simvastatin at 7 days as compared to the control group which consisted of simvastatin and chitosan alone. It was concluded that the topical application of simvastatin and chitosan gel could be used as a novel therapeutic modality that improved healing and reduced pain in the palatal donor site following the FGG procedure.

There are certain limitations noted in the current analysis such as (1) only 16 studies were included for the analysis based on a strict selection criterion, (2) studies had small sample sizes, and (3) different study designs which led to calibration error while documenting the results. However, the present review draws its strength from the strict selection bias and selection of the studies with conclusions drawn from the long duration of follow up.

## Conclusion

The data from the available studies suggest that simvastatin alone has a promising potential for alveolar bone regeneration in the optimal dose of 0.5–10 mg depending on the route of administration. The use of simvastatin with other bone grafts poses an additional advantage. However, there are contradictory results in literature; hence, more studies are needed for the same. A definitive consensus could not be reached on the use of carriers with simvastatin, and the usage of the carrier is determined by the site and type of surgical procedure. Despite positive outcomes for the healing of gingival graft, more clinical studies are needed to support the use of simvastatin for this application.
